# Periaortic Graft Infection Following Supracoronary Ascending Aorta Replacement: A Case Report

**DOI:** 10.7759/cureus.59914

**Published:** 2024-05-08

**Authors:** Issa Al-Khdour, Amro AlAqra, Moath Nairat, Nadine Yaghi

**Affiliations:** 1 Cardiac Surgery, An-Najah University Hospital, An-Najah National University, Nablus, PSE; 2 Cardiac Surgery, An-Najah National University Hospital, Nablus, PSE; 3 General Surgery, Faculty of Medicine, Al-Quds University, Jerusalem, PSE

**Keywords:** redo surgery, dissection, thoracic aortic graft infection, ascending aorta, adult cardiac surgery

## Abstract

Periaortic graft infections are a dangerous and extremely rare subtype of aortic graft infections (AGI). We hereby report a unique case of periaortic graft abscess in a 46-year-old male four months following a supracoronary ascending aorta replacement for DeBakey Type 2 dissection, resulting in the successful preservation of the original graft.

## Introduction

Aortic and periaortic graft infections are dreaded complications that can occur after aortic repair surgeries. Due to their rarity, with only a few reported cases worldwide according to a PubMed search, they still pose a significant challenge for surgeons, as their successful management entirely depends on prompt and accurate diagnosis to improve patient outcomes. Even with proper management, these infections still carry a high mortality rate [1]. There are currently no international guidelines that unanimously agree upon the preferred method of treatment, as they could be treated conservatively with a graft-preserving approach or surgically with the explantation of the infected graft [2]. We describe a 46-year-old male with a diagnosis of periaortic graft abscess who necessitated a redo sternotomy to evacuate the abscess and preserve the original graft.

## Case presentation

A 46-year-old male patient with a past medical history of chronic kidney disease presented to our hospital with the chief complaint of early-onset high-grade fever that persisted after a supracoronary ascending aorta replacement surgery with a synthetic graft at a governmental hospital four months ago. The surgery was uneventful at the time, but the patient developed early-onset fever four days after the operation and was discharged home on antibiotics for follow-up as a case of upper respiratory tract infection. His fever persisted despite being on multiple courses of antibiotics. After five weeks, he was transferred to our cardiothoracic surgery facility with the suspicion of aortic graft infection.

**Figure 1:**
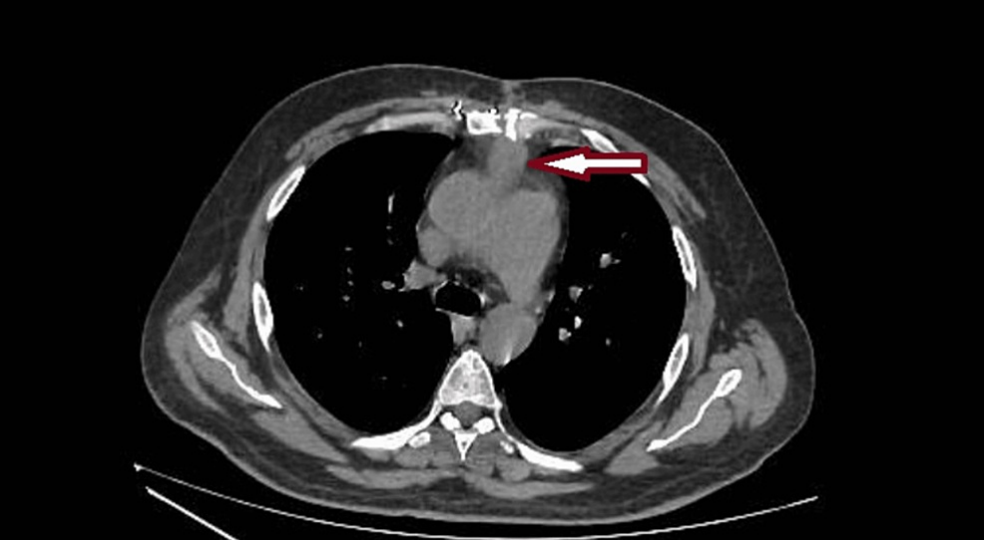
Chest CT demonstrating well-defined hypodensity (arrow) in the anterior mediastinum close to the anterior aspect of the ascending aorta as well as the posterior aspect of the sternum, with an appearance suggestive of loculated fluid

Upon admission, his laboratory studies were positive for elevated inflammatory markers, and blood cultures were collected and showed growth for Pseudomonas aeruginosa. Chest and abdomen CT without contrast demonstrated a well-defined hypodensity in the anterior mediastinum close to the anterior aspect of the ascending aorta, with an appearance suggestive of loculated fluid (Figure 1). Based on the collected findings, the patient was diagnosed with early-onset periaortic graft infection complicated by abscess formation. After a multidisciplinary team discussion with the interventional radiology department, it was decided to go for surgical evacuation and exploration of the mass.

**Figure 2:**
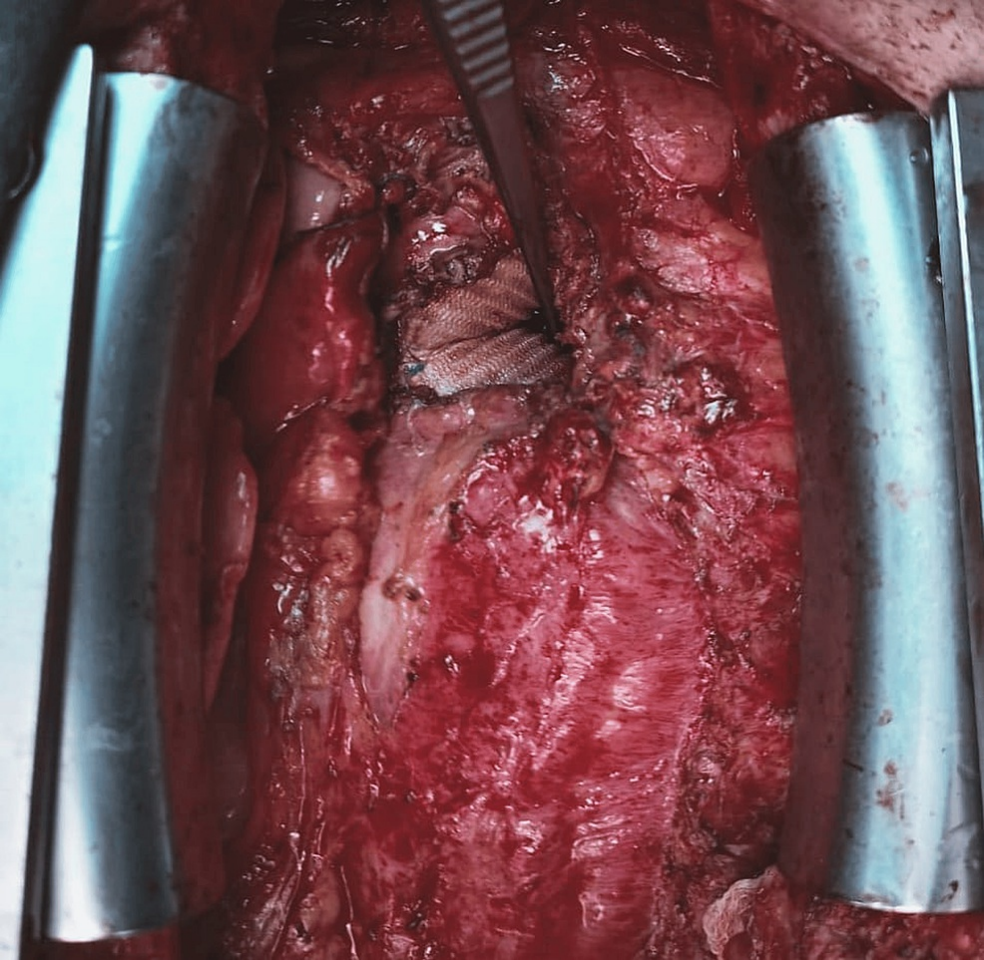
Intra-operative photo showing the intact aortic graft with the forceps placed in the area where the abscess pocket was found

The patient underwent a redo sternotomy where multiple small pockets with thick yellowish fluid were discovered intraoperatively within the area surrounding the ascending aorta, suggesting mediastinitis. The pockets were completely evacuated and excised. After extensive dissection, the abscess pocket was identified at the space between the aortic graft and the pulmonary artery (Figure 2). The abscess was completely evacuated, draining thick yellowish discharge, followed by wide debridement of the pocket and its surrounding tissue. The aortic graft was, however, found to be intact with no signs of fistulation or disruption. The infected area was then washed repeatedly with alcohol and saline followed by washing with vancomycin and gentamicin. Multiple cultures were collected intraoperatively that showed Pseudomonas aeruginosa growth.

**Figure 3:**
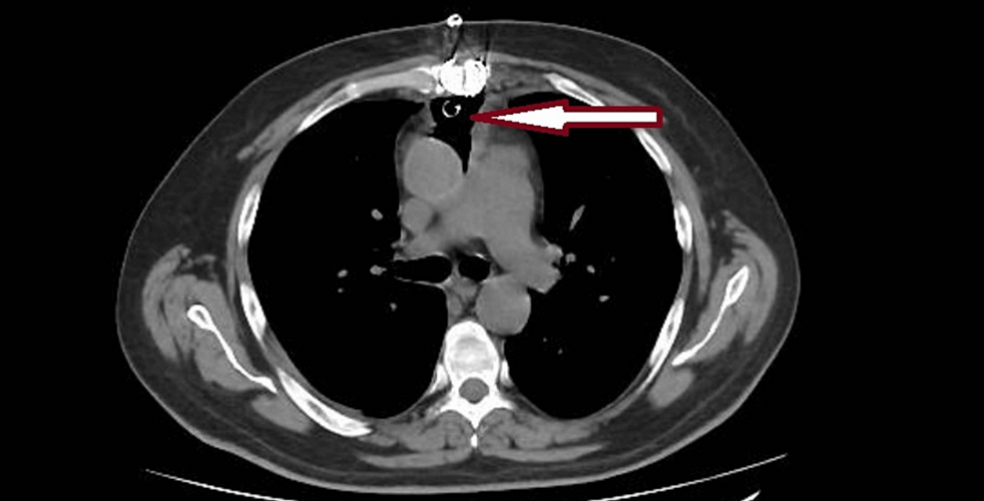
Postoperative chest CT demonstrating complete resolution of the loculated fluid from around the aorta (arrow)

At first, the management included intravenous antibiotics combined with irrigation therapy using both antibiotics and iodine. The patient’s clinical condition and his laboratory studies improved; however, he still had spikes of high-grade fever from time to time. In response to the persistent fever, we modified the antibiotics, and we replaced iodine irrigation with diluted alcohol irrigation. A 70% alcohol mixed with normal saline in equal volumes was given directly in the chest tube with a syringe under complete aseptic conditions, followed by clamping the irrigation system for two minutes, and then saline irrigation to ensure complete removal of the alcohol. This method was used twice a day for a week during which the patient’s fever resolved and the three fluid cultures retrieved from his chest tubes were negative as well as the rest of his septic workup. A postoperative chest CT scan confirmed the complete resolution of the previously detected collection around the aortic graft (Figure 3). The patient was discharged home on day 30, and he was maintained on ciprofloxacin for life.

## Discussion

Periaortic graft infections are catastrophic, rare complications that occur in a very small percentage, ranging from 0.2%-5%, following aortic repair surgeries [3]. Due to the scarcity of published literature on this matter, there are no well-known predisposing factors that are linked with aortic graft infections. However, the mortality rate, which ranges from 25% to 75%, renders them extremely vicious infections that must be promptly treated in order to save patient’s lives [1]. Post-aortic surgery graft infections can be subdivided into early-onset (less than four months) and late-onset infections (more than four months) [4]. Immediate postoperative infections are linked to direct inoculation of bacterial flora, most commonly Staphylococcus aureus, with a more abrupt toxic clinical presentation, while late-onset infections are associated with immunocompromised states, nosocomial septicemia, thrombosis, or postoperative leakage around the graft. However, late-onset infections are associated with less virulent pathogens, such as coagulase-negative Staphylococcus, Corynebacterium, or Propionibacterium, which explains the more indolent mild course of the disease [1,4]. Therefore, it is of paramount importance to recognize and identify the causative bacteria in cases of aortic graft infections, as medical treatment with antibiotics is imperative. Our patient presented with an early-onset postoperative infection that persisted due to improper handling and a low index of suspicion, which became complicated by bacteremia, which was in our case Pseudomonas aeruginosa.

Symptoms of aortic graft infections are considerably vague with nonspecific manifestations, ranging from high-grade fever, fatigue, weight loss, and abdominal and back pain, to nonspecific laboratory findings such as leukocytosis [5]. Therefore, a high index of suspicion must be present in order to diagnose this challenging complication that can manifest as any other infection.

The most reliable modality of choice in diagnosing aortic graft infections is computerized tomography (CT) with contrast enhancement [4]. Findings might include the following: persistence of perigraft fluid, presence of ectopic gas in the aortic wall, increased soft tissue with loss of normal tissue structure, and formation of pseudoaneurysms. If the thoracic aorta is suspected to be involved, CT can be supported by transthoracic echocardiogram (TTE) or transesophageal echocardiogram (TEE). In our case, CT contrast with contrast enhancement was the modality of choice for the diagnosis of the periaortic graft abscess, thus directing us to an effective strategy for management.

Although there are no clear guidelines on the diagnostic criteria and management of aortic graft infections, a study done by Lyons et al. in 2016 brought to light a diagnostic criterion called the management of aortic graft infection collaboration (MAGIC), which is based on clinical, radiological and laboratory findings, and sorts out each into three major and two minor criteria [4]. Based on this criterion, AGI is suspected in a patient with any single major criterion or two minor criteria from the three categories. AGI is confirmed, however, in the setting of a single major criterion, plus any other criterion whether major or minor from another category. Our case met two major laboratory and radiological criteria as well as one minor clinical criterion, confirming the diagnosis of AGI.

Yet, despite an attempt to formulate a universally accepted criterion for diagnosis, the study still faced limitations, as the literature lacks clinical-based evidence studies and more publications on this matter.

Regarding management, there is also no consensus on established treatment guidelines and there is a debate in the literature on the benefits of conservative versus surgical treatment. The modifications that were done by Samson et al. [6], as well as Koenig and vonDongen [7], over the wieldy used classification system for extra cavitary vascular graft infections established by Szilagyi et al. [8] have aided in establishing a treatment plan [9]. In the Samson class III group, graft preservation rather than graft reconstruction is recommended in early-onset infections, as was in our case, for better outcomes. Whereas in late-onset, Samson 3 suggests graft resection rather than graft preservation as it is more likely for the graft to be nonpatent and disrupted anastomoses [5]. Adjunct wound irrigation therapy with a povidone-iodine solution, which might be combined with antibiotics has also been suggested. In our patient, the addition of diluted alcohol to the irrigation therapy showed an accelerated clearance of fluid cultures obtained from the chest drains.

Antibiotic treatment with a broad gram-positive spectrum should be initiated until cultures are available, however, there is no specific duration for antibiotic use. Most published literature recommends a duration of treatment of four to six weeks while some studies suggest lifelong antibiotic treatment. In our case, the patient had comorbidities that contraindicated medical management alone not to mention that the bacterial growth in his blood was gram-negative [10]. This, in turn, explains the prolonged presentation of his symptoms that did not respond to the medical treatment that he was initially given over three months before being transferred to our hospital, for surgical debridement and evacuation was also indicated in his scenario after extensive evaluation on our part.

## Conclusions

Aortic and periaortic graft infections are fatal complications of aortic repair surgeries that necessitate timely intervention and accurate diagnosis based on surgical, radiological, and laboratory findings. Their high mortality rate depends on the time of the presentation, the extent of graft involvement, as well as the virulence of the pathogen strain involved. Surgical drainage and debridement in conjugation with culture-sensitive antibiotic use is the mainstay of treatment, often trying to preserve the original graft, which proved to be a successful approach in our patient. Despite the MAGIC criteria, there are still many unknown territories when it comes to the diagnosis and treatment modality of aortic graft infections and their subtypes. However, all the published literature agrees upon the significance of early diagnosis to prevent catastrophic complications, together with the importance of initiating empirical therapy as soon as there is an index of suspicion before collecting blood cultures. Conservative management alone, albeit effective in debilitating patients, carries a higher risk of mortality compared to combined surgical and medical treatment. Our case is one of a kind, will enrich the published literature on this deadly complication, and help broaden our understanding of effective management that should be tailored to every specific case according to its specific presentation. We faced particular difficulties with our patient, as his early-onset fever was managed as a late presentation.
